# Author Correction: Factors influencing riverine utilization patterns in two sympatric macaques

**DOI:** 10.1038/s41598-020-79259-1

**Published:** 2020-12-17

**Authors:** Yosuke Otani, Henry Bernard, Anna Wong, Joseph Tangah, Augustine Tuuga, Goro Hanya, Ikki Matsuda

**Affiliations:** 1 Center for the Study of Co* Design, Osaka University, Toyonaka, Osaka 560‑0043 Japan; 2 Institute for Tropical Biology and Conservation, Universiti Malaysia Sabah, Kota Kinabalu, Sabah Malaysia; 3 Forest Research Centre, Sabah Forestry Department, Sandakan, Sabah Malaysia; 4 Sabah Wildlife Department, Kota Kinabalu, Sabah Malaysia; 5 Primate Research Institute, Kyoto University, Inuyama, Aichi Japan; 6 Chubu University Academy of Emerging Sciences, Kasugai, Aichi Japan; 7 Wildlife Research Center of Kyoto University, Kyoto, Japan; 8 Japan Monkey Centre, Inuyama, Japan

Correction to: *Scientific Reports* 10.1038/s41598-020-72606-2, published online 25 September 2020

The original version of this Article contained an error in Affiliation 2, which was incorrectly given as ‘Institute for Tropical Biology and Conservation, University of Malaysia Sabah, Sabah, Malaysia’. The correct affiliation is listed below:

Institute for Tropical Biology and Conservation, Universiti Malaysia Sabah, Kota Kinabalu, Sabah, Malaysia

In addition, Reference 39 was incorrectly given as:

Matsuda, I. *et al.* The nose is mightier than the tooth: larger male proboscis monkeys have smaller canines. *bioRxiv*. 10.1101/848515 (2019).

The correct reference is listed below:

Matsuda, I. *et al.* Large male proboscis monkeys have larger noses but smaller canines. *Commun. Biol.* **3,** 522. 10.1038/s42003-020-01245-0 (2020).

These errors have now been corrected in the HTML and PDF versions of the Article.

